# Physicochemical modeling of the phytochrome-mediated photothermal sensing

**DOI:** 10.1038/s41598-019-47019-5

**Published:** 2019-07-19

**Authors:** Young-Joon Park, Chung-Mo Park

**Affiliations:** 1 Department of Chemistry, Seoul National University, Seoul, 08826 Korea; 2 Plant Genomics and Breeding Institute, Seoul National University, Seoul, 08826 Korea

**Keywords:** Light responses, Plant development

## Abstract

Light and temperature cues share many common signaling events towards plant photothermal morphogenesis. Particularly, the red (R)/far-red (FR)-absorbing phytochrome photoreceptors also function as temperature sensors, suggesting that light and temperature responses are intimately associated with each other. Here, we present data from physicochemical modeling of temperature sensing and thermomorphogenic patterning of hypocotyl growth, which illustrate that the two seemingly distinct stimulating cues are tightly coupled through physicochemical principles and temperature effects can be described as a function of infra-red (IR) thermal radiation. It is possible that the dark reversion from the FR-absorbing Pfr to the R-absorbing Pr phytochromes is essentially an IR-mediated thermal conversion. We propose that the phytochromes modulate photothermal responses by monitoring R:IR ratios, as they sense R:FR ratios during photomorphogenesis.

## Introduction

Plants are capable of actively sensing changes in light and temperature environments to optimize photothermal growth and morphogenesis. When we deal with the morphogenic effects of light in plants, light is usually taken to mean the visible spectrum, part of electromagnetic radiation. The effects of temperature on plant growth and environmental adaptation have been extensively studied at the molecular level. While light and temperature are assumed to be mutually distinct environmental signals in the field, accumulating evidence support that they share many common signaling mechanisms and events during plant photothermal responses^1–5^.

Notably, recent studies indicate that the red (R)/far-red (FR)-absorbing phytochrome photoreceptors also act as temperature sensors^3,4^. It has been reported that the dark-induced Pfr-to-Pr conversion is profoundly affected by temperature^3,4^. Along with the physicochemical nature of light and temperature, these observations suggest that light and temperature cues are intrinsically coupled through physicochemical principles in triggering the phytochrome-mediated photothermal responses.

Theoretically, molecules exhibit minimal movements at absolute zero temperature, and elevated temperatures accelerate molecular movements. In this regard, temperature is conceptually considered to be a measure of the average kinetic energy of the molecules. Molecules in rotational or vibrational movements emit or absorb energy in the form of infra-red (IR) radiation. Thus, all objects with temperatures above absolute zero, including the sun and plants in nature, emit electromagnetic radiation with distinct spectral distribution. In particular, the spectral distribution of thermal radiation depends solely on the temperature of the objects, necessitating that the spectral distribution of thermal radiation could reflect temperature changes.

In this work, to obtain clues as to the functional relationship between light and temperature cues in triggering the phytochrome-mediated photothermal behaviors, we employed physicochemical modeling analysis of environmental temperatures in a range of physiological relevance and thermomorphogenic examination of hypocotyl growth in *Arabidopsis* mutants that are defective in functional phytochromes or chromophore biosynthesis. Our physicochemical modeling and related calculations strongly support that the two apparently distinct environmental cues are intimately linked through physicochemical principles and the effects of environmental temperatures can be illustrated as a function of IR-mediated thermal radiation during the phytochrome-mediated photothermal responses. Notably, our data suggest that the Pfr-to-Pr dark reversion, which is recently proven to be accelerated by warm temperatures^3,4^, is virtually an IR conversion. On the basis of our data in conjunction with the photochemical properties of the phytochromes, we propose that the phytochromes constantly monitor fluctuations in R:IR ratios to modulate photothermal morphogenesis, which is comparable to the roles of the phytochromes in sensing R:FR ratios during photomorphogenesis.

## Results

### Spectral analysis of plant environmental temperatures

To explore whether it is plausible to consider plant environmental temperatures as thermal radiation, we employed the concept of spectral irradiance, which provides the power density of light with a particular wavelength. The spectral irradiance of electromagnetic radiation at a given temperature can be calculated according to the Planck’s law of black body radiation, which describes that a black body emits thermal radiation with distinct spectrum and intensity, entirely depending on its temperature. It has been shown that the spectral irradiance of solar radiation without discernible atmospheric absorption is very close to the theoretical value obtained from Planck’s law^6^. In addition, it has been reported that the emissivity of several plant species and other living organisms in their natural habitats is nearly close to 1^7^. We therefore reasoned that the black body assumption is readily applicable to the spectral analysis of plant environmental temperatures, which are in thermal equilibrium with plant body temperatures.

In the theoretical point of view, the black body is approximated to have an imaginary small hole such that radiation emitted from the hole represents a black body radiation. In the black body radiation, spectral energy density denotes spectral energy per unit volume, and irradiance indicates the flux received by a surface per unit area. When a specific temperature is given, spectral energy density and irradiance at the small hole of the black body can be theoretically obtained by Planck’s law (see Methods). Since spectral irradiance is inversely proportional to the square of distance, we are able to obtain the spectral irradiance of solar radiation on the surface of plants by considering the radius of the sun, the average radius of the earth’s orbit, and the absolute temperature of the sun.

**Figure 1:**
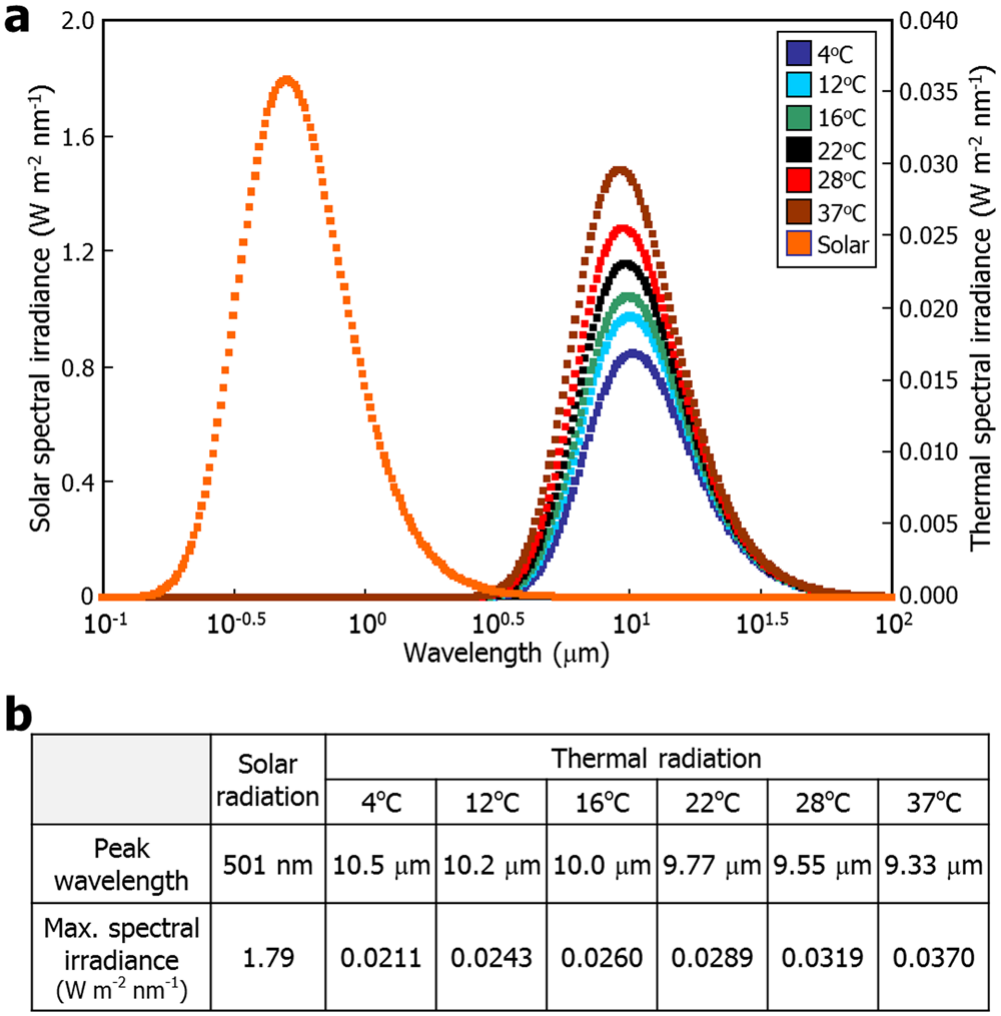
Spectral analysis of plant environmental temperatures. (**a**) Calculation of thermal spectral irradiance. Spectral irradiance of plant environmental temperatures in a range of 4 °C to 37 °C was calculated according to the Planck’s law of blackbody radiation. Solar radiation was included in the analysis for comparison. The calculation values were plotted against wavelength. The X-axis is represented in a logarithmic scale. (**b**) Peak wavelength of spectral irradiance. The peak wavelength and maximal spectral irradiance values were obtained from the spectral calculations in (**a**).

Spectral analysis according to Planck’s law showed that solar radiation had the peak wavelength of 501 nm (Fig. 1a,b and Supplemental Fig. 1a), as has been reported earlier^6^. We next analyzed the spectral irradiance of plant surfaces without solar irradiation so that we can examine exclusively environmental thermal radiation. In the spectral analysis of plant environmental temperatures, the distance factor goes to 1 because the imaginary spot on the plant surface is considered to be equivalent to the small hole of black body. Spectral analysis of plant environmental temperatures in a range of 4 to 37 °C, which are physiologically relevant in plant natural habitats, revealed that they had the peak wavelength of approximately 10 μm, which belongs to the IR spectrum range (Fig. 1a,b and Supplemental Fig. 1b). Data from the spectral analysis of thermal radiation is also in good agreement with the previous report that atmospheric absorption of light is relatively very low in a wavelength range of 8 to 14 μm within the overall IR spectral range^8^. It is thus reasonable to consider that IR light with the peak spectral irradiance at the wavelength of ~10 μm is effectively transmitted through the air to plants in nature.

### Plants emit IR thermal radiation in proportion to surrounding temperatures

**Figure 2:**
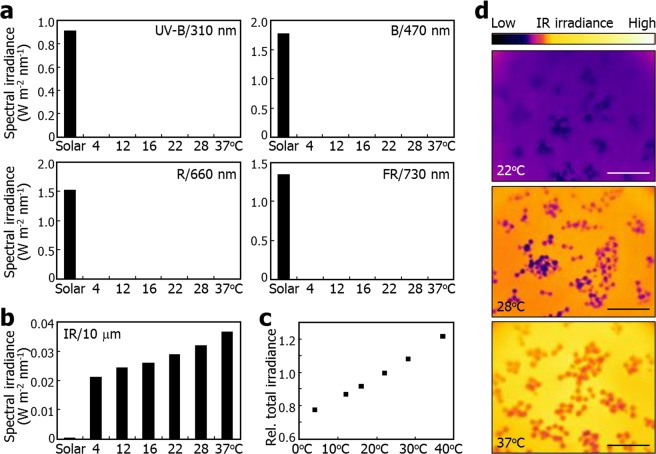
Plants emit IR thermal radiation in proportion to temperature. In (**a**) and (**b**), spectral irradiance values were plotted against environmental temperatures at different physiological wavelengths using the data from Table 1. (**a**) Spectral irradiance of thermal radiation in the UV-B and visible wavelength ranges. (**b**) Spectral irradiance of thermal radiation at 10 mm in the IR range. (**c**) Total spectral irradiance of thermal radiation. Total spectral irradiance was calculated by integrating the spectral irradiance values with respect to the wavelength range at each temperature given in Fig. 1a. (**d**) IR thermal imaging of *Arabidopsis* seedlings at different temperatures. Six-day-old seedlings were subsequently incubated at the indicated temperatures for 1 h. IR images were recorded in the wavelength range of 7.5~13 mm by a forward-looking IR camera. Scale bars = 20 mm.

We next analyzed the spectral irradiance of plant environmental temperatures at the specific wavelengths (Table 1). It was found that the spectral irradiance of plant environmental temperatures at the wavelength ranges of UV-B, blue (B), R, and FR lights were extremely low, and even negligible, in comparison to that of sunlight (Table 1 and Fig. 2a). On the other hand, the spectral irradiance of plant environmental temperatures at a wavelength of 10 μm were a hundred-fold higher than that of sunlight (Table 1).

**Table 1:** Thermal spectral irradiance at plant physiological wavelengths. Plants efficiently sense UV-B, blue (B), red (R), and far-red (FR) lights using distinct photoreceptors.

Light regime	Wavelength	Solar radiation (W m^−2^ nm^−1^)	Thermal radiation (W m^−2^ nm^−1^)					
			4 °C	12 °C	16 °C	22 °C	28 °C	37 °C
UV-B	310 nm	0.91	1.53 × 10^−65^	1.70 × 10^−63^	1.63 × 10^−62^	4.29 × 10^−61^	9.93 × 10^−60^	8.81 × 10^−58^
B	470 nm	1.77	1.08 × 10^−41^	2.43 × 10^−40^	1.08 × 10^−39^	9.37 × 10^−39^	7.47 × 10^−38^	1.45 × 10^−36^
R	660 nm	1.52	2.27 × 10^−28^	2.06 × 10^−27^	5.93 × 10^−27^	2.74 × 10^−26^	1.19 × 10^−25^	9.70 × 10^−25^
FR	730 nm	1.35	1.44 × 10^−25^	1.08 × 10^−24^	2.82 × 10^−24^	1.14 × 10^−23^	4.35 × 10^−23^	2.95 × 10^−22^
IR	10 μm	2.86 × 10^−4^	0.0210	0.0243	0.0260	0.0288	0.0318	0.0366

They are also responsive to IR thermal radiation. Spectral irradiance was calculated at the specific wavelengths. The wavelength of IR was set to 10 μm, around which the spectral irradiance of thermal radiation was maximal in Fig. 1.

It is particularly interesting that the spectral irradiance steadily increases in proportion to the elevation of environmental temperatures (Fig. 2b). In addition, total irradiance along all the light wavelength range similarly increases as plant environmental temperatures rise (Fig. 2c). The direct proportion between total irradiance and temperatures is in good accordance with Stefan-Boltzmann law that energy radiated from unit surface area per unit time is proportional to the fourth power of temperature. Together, these physicochemical data support that plant environmental temperatures in fact represent IR thermal radiation.

In order to validate the physicochemical basis for considering plant environmental temperatures as IR thermal radiation, we directly monitored IR irradiance of plants following dark incubation at different temperatures using a forward-looking IR camera with an optical capacity of detecting light wavelength in a range of 7.5 to 13 μm. It was found that IR thermal irradiance, as accessed by IR thermal imaging, evidently increases as plant environmental temperatures go up (Fig. 2d). This observation is well consistent with the notion that plant environmental temperatures are represented as IR thermal radiation.

### IR thermal radiation would underlie the dark reversion of the phytochromes

A critical question was which photoreceptors are responsible for sensing IR thermal radiation. The R/FR-absorbing phytochrome photoreceptors are known to act as temperature sensors^3,4^. In particular, our spectral analysis predicted that sensing temperatures is physicochemically linked with IR responsiveness (Fig. 1). Thus, it was anticipated that the phytochrome photoreceptors mediate the responsiveness to IR thermal radiation.

The phytochromes exist in two spectrally interconvertible forms: the R-absorbing Pr form and the FR-absorbing Pfr form, which is attributed to the *cis-trans* photoisomerization of the phytochromobilin (PQB) chromophore. Pr is readily converted to Pfr upon R absorption^9^. Inversely, Pfr is converted to Pr upon FR illumination. Notably, the Pfr-to-Pr conversion also occurs spontaneously in the dark, a photochemical process frequently called dark reversion^10,11^. While the Pfr-to-Pr dark reversion has been noticed for several decades, it remains elusive how the photoactivated status of the photoreceptors change under dark conditions. Meanwhile, it is well known that experimental dark conditions routinely used in ordinary laboratories or under natural conditions do not represent absolute darkness because all the objects above absolute zero temperature emit IR thermal radiation. Thus, we hypothesized that the dark reversion process of the phytochromes would be associated with their responsiveness to IR thermal radiation.

To examine whether the Pfr-to-Pr dark reversion is affected by IR thermal radiation, we slightly modified the theoretical model of the Pfr kinetics^12^, which considers only the photoconversion events by R and FR lights. Since dark reversion is accelerated in response to warm temperatures, we accordingly employed an additional term that represents the contribution of IR to the equation of the Pfr kinetics (see Methods). The resultant modified model is able to estimate the contributions of R, FR, and IR lights to the Pfr dynamics. The photon flux of R and FR lights is zero during dark reversion. Thus, it is apparent that the Pfr kinetics during dark reversion is modulated entirely by IR thermal radiation.

**Figure 3:**
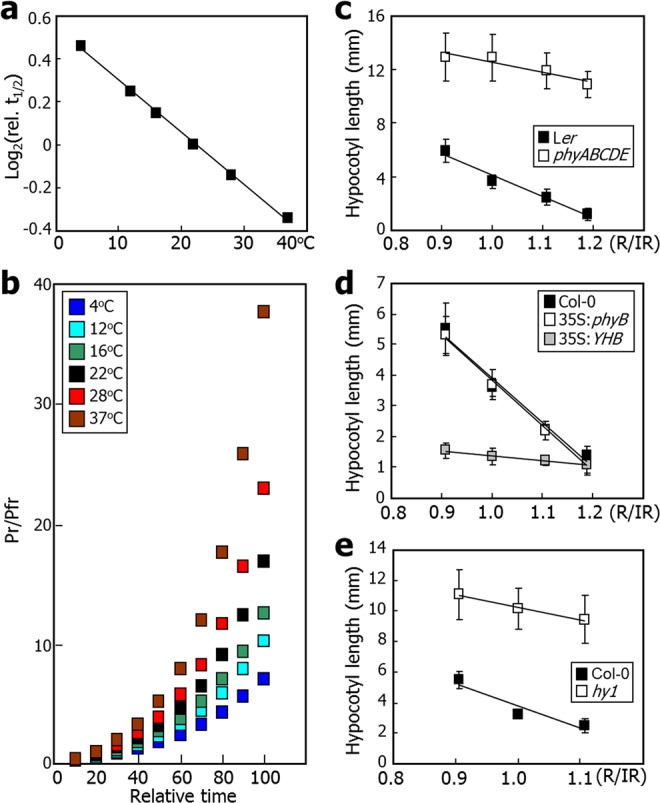
IR thermal radiation influences the phytochrome-mediated photothermal morphogenesis. (**a**) Thermal kinetics of the Pfr-to-Pr conversion. The IR spectral irradiance values in Table 1 were used to calculate the half-life (t_1/2_) of Pfr at different temperatures. The t_1/2_ of Pfr at 22 °C was set to 1. (**b**) Estimation of Pr/Pfr ratios during dark reversion. (**c**) IR control of the *phyABCDE* hypocotyl growth. The *Arabidopsis* seedlings were grown under short days for 7 d at different temperatures. The R/IR ratio at 22 °C was set to 1. (**d**) IR-insensitive hypocotyl phenotype of *Arabidopsis* seedlings overexpressing a constitutively active phyB (YHB). Seedlings were grown as described in (**c**). The R/IR ratio at 22 °C was set to 1. (**e**) Effects of IR radiation on the hypocotyl growth of chromophore-defective *hy1* mutant. Seedlings were grown under short days for 7 d at different temperatures. The R/IR ratio at 22 °C was set to 1.

Our physicochemical modeling through the spectral analysis of plant environmental temperatures showed that the relative half-life of Pfr in the dark is inversely proportional to temperature elevation in a range of 4 °C to 37 °C [Log_2_ (relative *t*_1/2_) = −0.0243*T* + 0.5435, where *T* is temperature (°C) and relative *t*_1/2_ at 22 °C = 1, *R*^2^ = 0.9988] (Fig. 3a). The half-life estimations were also consistent with the temperature-dependent regulation of the half-life of Pfr *in vivo*^3,4^.

On the basis of our physicochemical modeling, we also plotted the kinetics of Pr/Pfr ratios during dark reversion (see Methods). It was found that the IR-mediated alterations of Pr/Pfr ratio is exponentially proportional to the time period of dark incubation (Fig. 3b). These results are in good accordance with the *in vivo* measurements of phytochrome dynamics in etiolated seedlings^3^. Thus, it is evident that the dark reversion of Pfr is associated with the responsiveness of the phytochromes to IR thermal radiation. In addition, it is likely that the thermal acceleration of dark reversion is caused by increasing IR irradiance. In this regard, it would be more plausible to consider the Pfr-to-Pr conversion in the dark as IR conversion.

### The phytochromes modulate the IR-mediated thermal control of plant morphogenesis

We next asked whether the regulation of plant thermomorphogenesis by IR thermal radiation is mediated by the phytochromes. The *Arabidopsis phyABCDE* mutant lacking functional phytochromes were grown at varying temperatures, and hypocotyl lengths were compared to those of control seedlings grown under identical conditions. We calculated a relative R:IR ratio at each temperature according to our spectral analysis of IR thermal radiation (see Methods). It was found that the hypocotyl lengths of control seedlings showed a strong negative linear relationship against increasing R:IR ratios [*h* = −16.177*r* + 20.27, where *r* is the relative R:IR ratio and relative *r* at 22 °C = 1, *R*^2^ = 0.973] (Fig. 3c). In contrast, those of the *phyABCDE* mutant seedlings were widely deviated from a linear relationship, and their sensitivity to increasing R:IR ratios was significantly reduced [*h* = −7.2727*r* + 19.762, *R*^2^ = 0.8791]. These observations are consistent with the IR-mediated control of the phytochrome function in the regulation of hypocotyl growth.

It has been reported that phyB harboring the Y276H substitution, termed YHB, behaves as a constitutively active Pfr form and transgenic plants expressing YHB are insensitive to ambient temperatures^3,13–16^. Measurements of hypocotyl growth in seedlings grown at different temperatures in the light showed that the hypocotyl lengths of 35 S:*phyB-GFP* transgenic seedlings, which overexpress a phyB-GFP fusion driven by the strong CaMV 35 S promoter, exhibit a negative linear relationship against increasing R:IR ratios [*h* = −14.489*r* + 18.397, where *r* is the relative R:IR ratio and relative *r* at 22 °C = 1, *R*^2^ = 0.9787], similar to what observed with Col-0 seedlings [*h* = −14.879*r* + 18.705, *R*^2^ = 0.9965] (Fig. 3d). On the other hand, the hypocotyl growth of 35 S:*YHB-GFP* transgenic seedlings were virtually insensitive to varying R:IR ratios [*h* = −1.5748*r* + 2.9523, *R*^2^ = 0.9889], supporting the role of the phytochromes in regulating hypocotyl growth in response to IR thermal radiation.

The Pr-Pfr photoconversion is mediated by the PQB chromophore, which undergoes a *cis*-*trans* isomerization upon R/FR light stimulation^9,17^. Heme oxygenase (HO) enzymes mediate the oxidative cleavage of the heme ring to synthesize biliverdin IXα, the committed step towards the biosynthesis of the phytochrome chromophores^18,19^. The *Arabidopsis hy*1 mutant, which is defective in chromophore biosynthesis^20–22^, was subjected to photothermal analysis. It was found that unlike the prominent effects of varying R:IR ratios on Col-0 seedling growth [*h* = −14.853*r* + 18.662, where *r* is the relative R:IR ratio and relative *r* at 22 °C = 1, *R*^2^ = 0.9036], the effects of R:IR ratios were slightly reduced in the *hy1* seedling growth [h = −8.2234*r* + 18.489, *R*^2^ = 0.9880] (Fig. 3e). These observations allow us to propose that it is conceptually plausible that the PQB chromophore is important for the responsiveness of the phytochromes to IR thermal radiation, further supporting that IR thermal radiation influences the phytochrome-mediated thermal control of hypocotyl growth.

Altogether, our data from physicochemical modeling of temperature and photothermal analysis of hypocotyl growth suggest that the phytochromes mediate the thermal control of plant morphogenesis by sensing environmental temperatures in the form of IR thermal radiation. The physicochemical correlationship between light and temperature is also applicable to the Pfr-to-Pr dark reversion, and the dark reversion would be conceptually described as IR conversion, similar to the R and FR conversion events during the well-known photoconversion of the phytochromes.

## Discussion

The PQB chromophore is covalently attached to the phytochrome apoprotein via a highly conserved cysteine residue in higher plants^23–25^. PQB is a linear tetrapyrrole bilin, which mediates the photoconversion between two spectrally distinct Pr and Pfr forms^9^. In the Pfr phytochrome, PQB has a *trans* configuration, whereas it has a *cis* configuration in the Pr phytochrome (Supplemental Fig. 2a).

Interestingly, some organic compounds have been shown to undergo a photothermally induced isomerization process, similar to the photothermal conversion of the phytochromes^26^. For example, azobenzene is readily converted from the *trans* form to the *cis* form in response to light of short wavelength, such as UV, while the reversion to the *trans* form is temperature-dependent^26^ (Supplemental Fig. 2a). It is therefore feasible that the chromophore plays a key role in the ability of the phytochromes to respond to IR thermal radiation as well as R and FR lights.

**Figure 4:**
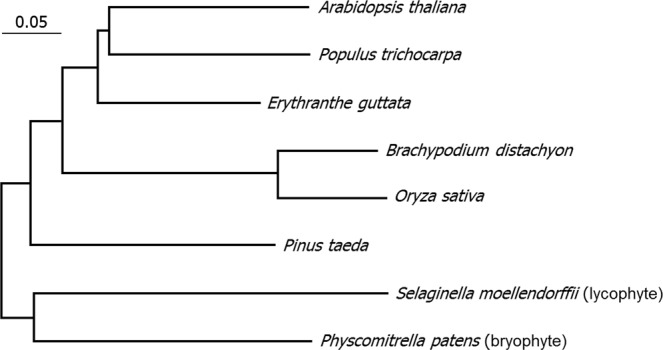
Conservation of heme oxygenase 1 (HO1) enzymes in land plants. HO enzymes mediate the oxidative cleavage of the heme ring to produce biliverdin IXα, the committed step towards the biosynthesis of the phytochrome chromophores, such as PQB. Public database searches identified HO1 enzyme genes from a wide array of plant species, including the earliest land plants (bryophyte and lycophyte). Amino acid sequences of the HO1 proteins were retrieved from the NCBI database (http://www.ncbi.nlm.nih.gov/protein). A phylogenetic tree was constructed using the MEGA7 software with the neighbor-joining method.

Notably, phylogenetic analysis of HO enzymes, which mediate the committed step in chromophore biosynthesis, revealed that they are highly conserved among a broad spectrum of plant species, including the earliest land plants, bryophytes and lycophyte (Fig. 4). Together with the critical role of the PQB chromophore for the IR responsiveness of the phytochromes, this observation suggests that the IR responsiveness of the chromophore has an ancient origin, perhaps up to the evolutional origin of land plants from aquatic life.

While the aquatic-to-terrestrial transition likely brought some adaptive advantages on plants, such as higher radiation of sunlight and aerial carbon dioxide, terrestrial life would have imposed some challenges, such as abrupt temperature fluctuations. To cope with these newly imposed challenges, land plants have evolved diverse morphological and photothermal devices, such as the temperature-sensing phytochromes^27,28^. It is anticipated that the acquisition of the PQB chromophore by land plants would help the phytochromes respond more efficiently to temperature changes. Thus, it will be interesting to compare the IR responsiveness of PQB with that of phycoerythrobilin (PEB) and phycocyanobilin (PCB) chromophores.

A number of biochemical and photochemical studies has been carried out to understand the photoconversion process of the phytochromes, and physiological models depicting the phytochrome actions are emerging in recent decades^29–33^. It has been extensively studied how the photoactivated status of the phytochromes is modulated by light fluence. Notably, multiple intramolecular and intermolecular features allosterically influence the Pfr status by modulating the dimerization of the phytochrome molecules and their interactions with downstream signaling mediators^29,31^.

Our physicochemical modeling describes mainly the contribution of IR radiation to the phytochrome action under warm conditions, where IR radiation is prominent. For example, hypocotyl thermomorphogenesis occurs mainly at the end of the night under short days^2,3,34^, which is in harmony with the accelerated IR reversion at warm nights. On the basis of the previous and our own data, it is proposed that our model on the role of IR thermal radiation is physiologically relevant for the regulation of hypocotyl thermomorphogenesis. It will be interesting to investigate whether and how the IR-mediated responses proposed in this study are interrelated with IR-independent mechanisms, such as phytochrome dimerization, allosteric interactions, and photobody formation^4,29–33^, which are also known to modulate the phytochrome function.

A previous report has shown that the temperature-dependent dark reversion of the phytochromes occurs at least for 8 hours following temperature treatments in 4-day-old seedlings^3^. In addition, our modeling suggests that the rate of IR-mediated Pfr-to-Pr conversion is exponentially proportional to the time period during dark reversion. Since IR thermal radiation increases as temperature increases, the effects of IR seem to be dosage-dependent. On the basis of our model and the previous reports of phytochrome-mediated temperature sensing during the night, we believe that the effects of IR are sustained over a warm night.

**Figure 5:**
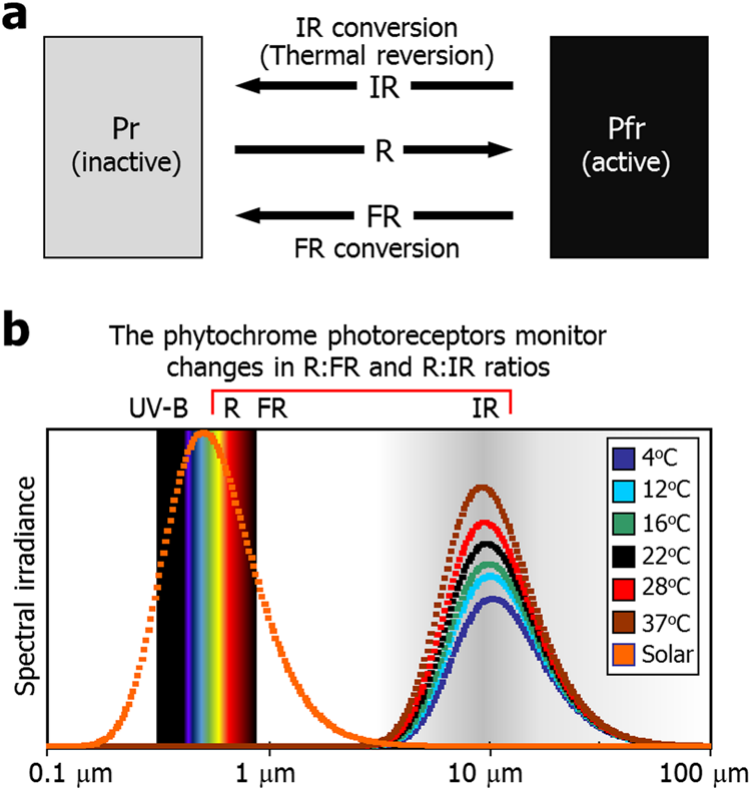
The phytochromes are versatile photothermal receptors that are capable of responding to changes in R:FR and R:IR ratios. (**a**) Physicochemical modeling for the photothermal conversion of the phytochromes. (**b**) Perception of R:FR and R:IR ratios during photothermal morphogenesis. The phytochromes sense environmental temperatures in the form of IR thermal radiation in a similar physicochemical process as they do R and FR during photomorphogenesis. The X-axis is represented in a logarithmic scale.

Our data obtained from physicochemical modeling analysis, in addition to molecular biological and physiological tools, suggest that the phytochromes sense environmental temperatures as IR thermal radiation and monitor changes in R:IR ratio to regulate photothermal morphogenesis (Fig. 5a). It is now evident that the phytochromes are responsive to a broader spectrum of light information than previously thought^35,36^ (Fig. 5b). The physicochemical modeling approaches also explain why the Pfr-to-Pr conversion occurs even in the dark in a temperature-dependent manner. Our data further extend the repertoire of the phytochrome functions: the phytochrome photoreceptors monitor changes in R:IR ratio to coordinate photothermal responses under fluctuating temperature conditions, as they modulate photomorphogenic responses in response to changes in R:FR ratio.

## Methods

### Spectral analysis of thermal radiation

We employed the Planck’s law of black body radiation to explore the physicochemical characteristics of thermal radiation. This physicochemical principle illustrates the spectral density of electromagnetic radiation emitted from a black body that remains in thermal equilibrium with its environment at a specific temperature. According to Planck’s law, the spectral energy density ρ_*T*_ (*λ*) of thermal radiation is given by

$${{\rm{\rho }}}_{T}(\lambda )=\frac{8\pi hc}{{\lambda }^{5}}\frac{1}{{e}^{hc/\lambda {k}_{B}T}-1}$$

where *h* is Planck’s constant with a value of 6.626 × 10^−34^ J·s, *c* is the speed of light with a value of 2.998 × 10^8^ m·s^−1^, *λ* is the wavelength in m, *k*_*B*_ is Boltzmann’s constant with a value of 1.381 × 10^−23^ J·K^−1^, and *T* is the absolute temperature of the object in K.

The irradiance *E*, the flux received by a surface per unit area, is given by *E* = *c* ·*ρ*. Black body assumption includes the isotropic diffusion of thermal radiation. With this assumption, the plane irradiance *E*_*d*_ is given by *E*_*d*_ = *E*/4, where *E* is total irradiance. Collectively, the spectral plane irradiance is given by

$${E}_{dT}(\lambda )=\frac{2\pi h{c}^{2}}{{\lambda }^{5}}\frac{1}{{e}^{hc/\lambda {k}_{B}T}-1}$$

Since the irradiance is inversely proportional to distance, the spectral irradiance of solar radiation can be transformed into

$${E}_{d{\rm{solar}}}(\lambda )={(\frac{{R}_{{\rm{sun}}}}{{R}_{{\rm{orbit}}}})}^{2}\frac{2\pi h{c}^{2}}{{\lambda }^{5}}\frac{1}{{e}^{hc/\lambda {k}_{B}{T}_{{\rm{sun}}}}-1}$$

where *R*_sun_ is the radius of the sun with a value of 6.95 × 10^8^ m, *R*_orbit_ is the average radius of the earth’s orbit with a value of 1.50 × 10^11^ m, and *T*_sun_ is the absolute temperature of the sun with a value of 5778 K. It has been reported that solar radiation is close to the radiation of black body^6^.

To calculate the thermal radiation of plant environmental temperatures, we assumed that environmental temperatures are in thermal equilibrium with plant body temperatures such that the distance factor goes to 1. Then, the spectral irradiance of plant environmental thermal radiation is given by

$${E}_{d{\rm{environment}}}(\lambda )=\,\frac{2\pi h{c}^{2}}{{\lambda }^{5}}\frac{1}{{e}^{hc/\lambda {k}_{B}{T}_{{\rm{environment}}}}-1}$$

where the absolute temperature of plant environmental temperatures *T*_environment_ = 277.15, 285.15, 289.15, 295.15, 301.15, 310.15, and 310.15 K for 4, 12, 16, 22, 28, and 37 °C, respectively, which are physiologically relevant in plant natural habitats. The emissivity of several plant species and other living organisms is nearly close to 1^7^, supporting that black body assumption is readily applicable to our physicochemical model. Total irradiance was calculated by integrating spectral irradiance values with respect to wavelength, which is also known as Stefan-Boltzmann law.

### Plant materials

*Arabidopsis thaliana* lines used were in Columbia (Col-0) background except for the *phyABCDE* mutant, which has been generated in L*er* background^37^. The 35 S:*phyB-GFP* transgenic plants in Col-0 background have been described previously^38^. The 35 S:*YHB-GFP* transgenic plants overexpress a modified *phyB* gene that encodes a constitutively active phyB (YHB)^16^. The YHB protein harbors the Y276H substitution^13^. The loss-of-function *hy1-100* mutant was isolated from a pool of mutant lines deposited in the *Arabidopsis* Biological Resource Center (Ohio State University, Columbus, OH).

### Infrared thermography

Seedlings grown at 23 °C for 6 days under long days (16-h light and 8-h dark) were subsequently incubated at different temperatures for 1 h in the dark. The infrared images of the temperature-treated seedlings were recorded using the thermal imaging camera T420 (FLIR, Wilsonville, OR), as described previously^39^. The infrared images were then analyzed using the FLIR Tools (http://www.flirkorea.com/home).

### Physicochemical modeling of IR-mediated thermal reversion

To examine whether the Pfr-to-Pr dark conversion can be explained by IR responsiveness, we employed a model of Pfr kinetics. The phytochrome photoconversion is affected by the extinction coefficient (*ε*) of the pigment (chromophore) and the quantum yield (*ϕ*) of the process, where the photoconversion cross-section (*σ*) of the pigment is given by 2.3 × *ε ϕ*^12^. In a phytochrome solution exposed to a thermal radiation of photon flux *N*_*λ*_, the rate of change in [Pfr] is given as follows:

$$\frac{d[{\rm{Pfr}}]}{dt}={N}_{R}{\sigma }_{R}[{\rm{\Pr }}]-{N}_{{\rm{FR}}}{\sigma }_{{\rm{FR}}}[{\rm{Pfr}}]-{N}_{{\rm{IR}}}{k}_{{\rm{IR}}}[{\rm{Pfr}}]$$

where the coefficient *k*_IR_ includes the efficiency of IR thermal radiation on the Pfr-to-Pr conversion. It is evident that *N*_R_ = 0 and *N*_FR_ = 0 because there is no visible spectrum during dark reversion. Therefore, the rate of change in [Pfr] during dark reversion is given as follows:

$$\frac{d[{\rm{Pfr}}]}{dt}=-\,{N}_{{\rm{IR}}}{k}_{{\rm{IR}}}[{\rm{Pfr}}]$$

As a result, $\mathrm{ln}\,{[{\rm{Pfr}}]}_{t}=\,\mathrm{ln}\,{[{\rm{Pfr}}]}_{{t}_{0}}-{k}_{{\rm{IR}}}{\sigma }_{{\rm{IR}}}t\,$, where [Pfr]_t_ is the amount of Pfr at time t, and [Pfr]_t0_ is the initial amount of Pfr at time t_0_.

From the above equation, the half-life of Pfr t_1/2_ is given by $\frac{\mathrm{ln}\,2}{{N}_{{\rm{IR}}}{k}_{{\rm{IR}}}}$. The difference of half-life could be described as follows:

$$\mathrm{log}\,{t}_{1/2}({T}_{1})-\,\mathrm{log}\,{t}_{1/2}({T}_{2})=\,\mathrm{log}\,\frac{{N}_{{\rm{IR}}}({T}_{2})}{{N}_{{\rm{IR}}}({T}_{1})}$$

where *T*_*1*_ and *T*_2_ denote two different plant environmental temperatures. The half-life of Pfr t_1/2_ at 22 °C was set to 1, and the relative half-life values of Pfr at varying plant environmental temperatures were calculated. The IR photon flux *N*_IR_ was calculated from the spectral irradiance of plant environmental thermal radiation with the wavelength of 10 μm.

To plot the kinetics of the Pfr-to-Pr conversion during dark reversion, the differential equation was integrated, resulting in the following formula

$$[{\rm{Pfr}}]={[{\rm{Pfr}}]}_{t0}\cdot {e}^{-{N}_{{\rm{IR}}}{k}_{{\rm{IR}}}t}$$

The ratio of Pr/Pfr could be described as follows:

$$\frac{[{\rm{\Pr }}]}{[{\rm{Pfr}}]}=\frac{{[{\rm{Pfr}}]}_{t0}-[{\rm{Pfr}}]}{[{\rm{Pfr}}]}={e}^{{N}_{{\rm{IR}}}{k}_{{\rm{IR}}}t}-1$$

where *t* is the incubation time in the dark. Since *N*_IR_ and *k*_IR_ are constants, we plotted the Pr/Pfr ratios against relative time period.

### Measurements of hypocotyl length

*Arabidopsis* seeds were sterilized and sown on 1/2 X Murashige and Skoog-agar plates (hereafter, referred to as MS-agar plates). The sterilized seeds were incubated at 4 °C for 3 days for cold stratification. They were then allowed to germinate and grow in a temperature-controlled culture room set at 22 °C with relative humidity of 55% under short days (8-h light and 16-h dark). White light illumination (120 μmol photons m^−2^ s^−1^) was provided by fluorescent FLR40D/A tubes (Osram, Seoul, Korea). The seedlings were then transferred to various temperature conditions. Seven-day-old seedlings were photographed, and hypocotyl lengths were measured using the ImageJ software^40^. Fifteen plants were analyzed for each measurement, unless otherwise specified.

### Calculation of R:IR ratio

Relative R:IR ratios were calculated according to the spectral analysis data in Fig. 1 and Table 1. Under the assay conditions employed, while light intensity is constant, temperature varies. When seedlings are exposed to a spectral radiation of wavelength *λ* and photon flux *N*_*λ*_, the relative R:IR ratio is given as follows:

$$r({T}_{1},{T}_{2})=\frac{\frac{{N}_{R}}{{N}_{{\rm{IR}}}({T}_{2})}}{\frac{{N}_{R}}{{N}_{{\rm{IR}}}({T}_{1})}}=\frac{{N}_{{\rm{IR}}}({T}_{1})}{{N}_{{\rm{IR}}}({T}_{2})}$$

where IR photon flux *N*_IR_ was calculated from the spectral irradiance of plant environmental thermal radiation at the wavelength of 10 μm.

### Phylogenetic analysis

The amino acid sequences of HO1 enzymes were inferred from the genomic sequences of *Arabidopsis thaliana*, *Populus trichocarpa*, *Erythranthe guttata*, *Brachypodium distachyon*, *Oryza sativa*, *Pinus taeda*, *Selaginella moellendorffii*, and *Physcomitrella patens* deposited in the NCBI database (http://www.ncbi.nlm.nih.gov/protein). *Selaginella moellendorffii* and *Physcomitrella patens* are considered as the earliest land plants^41,42^. The phylogenetic tree was constructed using the MEGA7 software with the neighbor-joining method^43^.

## Supplementary information

Supplementary Information

## Data Availability

All data generated or analyzed during this study are included in this article and its Supplementary Information Files.
