# Supplementary material for: Physicochemical modeling of the phytochrome-mediated photothermal sensing
Source: Sci Rep. 2019 Jul 19;9:10485. doi: 10.1038/s41598-019-47019-5 (PMC6642129; doi:10.1038/s41598-019-47019-5)
Supplement: Supplementary file 1 — Supplementary Information [file 41598_2019_47019_MOESM1_ESM.pdf]

Physicochemical modeling of the phytochrome-mediated photothermal sensing

Young-Joon Park, Chung-Mo Park

Supplemental Fig. 1

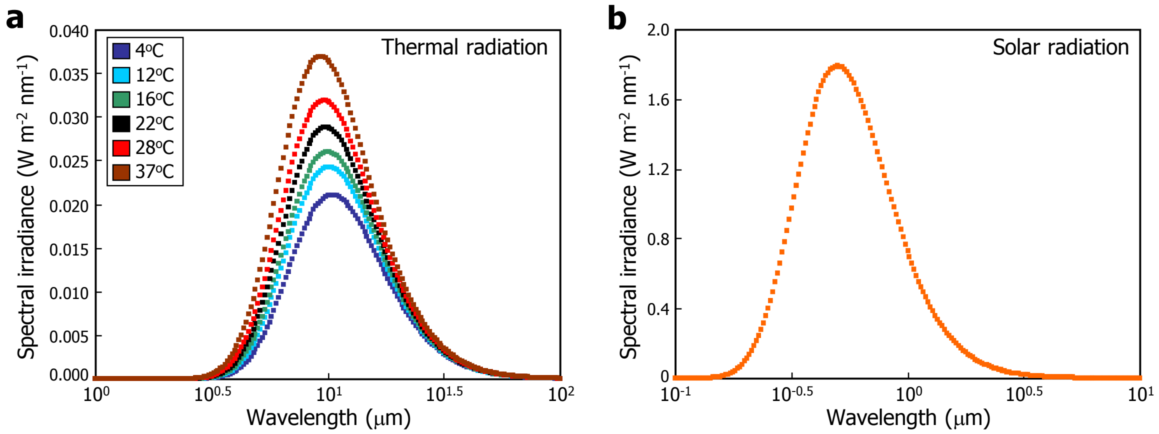

Supplemental Figure 1. Temperature changes provoke the emission of infra-red thermal radiation. Spectral analysis was performed on plant environmental temperatures in a range of 4ºC to 37ºC, which are important for plant growth and adaptation in nature. Spectral irradiance was calculated according to the Planck's law of blackbody radiation. Solar radiation was included in the analysis for comparison. The spectral irradiance curves for thermal radiation and solar radiation (**a** and **b**, respectively) were combined in Fig. 1a. The X-axis is represented in a logarithmic scale in the graphs. (**a**) Spectral irradiance curve for thermal radiation. (**b**) Spectral irradiance curve for solar radiation.

Supplemental Fig. 2

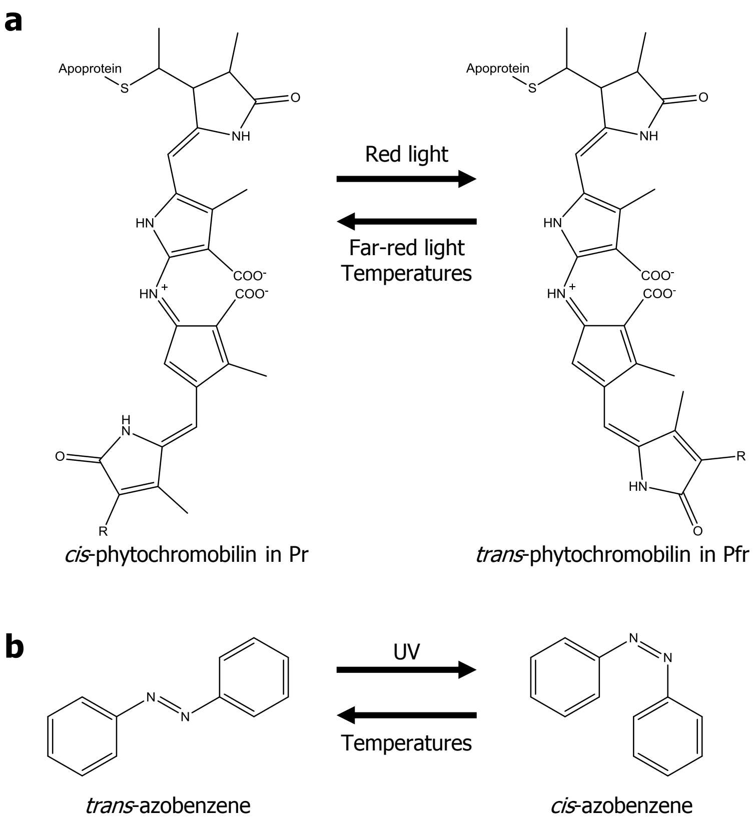

Supplemental Figure 2. Reversible photothermal isomerization of the phytochrome chromophore. (**a**) Photothermal control of the phytochromobilin isomerization. The *cis* configuration of phytochromobilin in the Pr phytochrome is photoconverted to the *trans* configuration in the Pfr phytochrome by red light illumination. The *trans*-phytochromobilin in the Pfr phytochrome is photoconverted back to the *cis*-phytochromobilin in the Pr phytochrome by far-red light illumination. The *trans*-to-*cis* isomerization also occurs in the dark, thus termed dark reversion. Notably, the dark reversion is accelerated by thermal radiation. (**b**) The photothermal isomerization of azobenzene. The *trans*-azobenzene is converted to the *cis*-azobenzene in response to UV light. The *cis*-azobenzene is converted back to the *trans*-azobenzene by thermal radiation.
